# *Aroui minusetosus*, a new species of Scopelocheiridae from Korea (Crustacea, Amphipoda, Lysianassoidea)

**DOI:** 10.3897/zookeys.706.20007

**Published:** 2017-10-04

**Authors:** Tae Won Jung, Charles Oliver Coleman, Seong Myeong Yoon

**Affiliations:** 1 Museum für Naturkunde Berlin, Berlin 10115, Germany; 2 Department of Biology, Chosun University, Gwangju 16452, Korea

**Keywords:** *Aroui minusetosus* sp. n., amphipod, Korea, lysianassoid, Scopelocheiridae, taxonomy

## Abstract

A new species, *Aroui minusetosus* **sp. n.**, is recorded from Korean waters with detailed description and illustrations. A new key to all known *Aroui* is provided. The Korean material of this scopelocheirid is readily assigned to the genus *Aroui* by the presence of long and distally barbed setae on the outer plate of maxilla 2. This new species is distinguished from congeners by ventrally smooth coxae 1-3 and a setose posteroventral margin of coxa 4, the subchelate gnathopod 2 having a row of four robust setae on its posterior margin (including an elongate single locking seta), and the setation of all appendages which is less dense than in other species. This is the first record of scopelocheirid amphipods from Korean waters.

## Introduction

The family Scopelocheiridae was established by Lowry and Stoddart (1997) based on the synapomorphy of a strongly shortened dactylus of gnathopod 1 which is covered with various distal setae from the propodus. Simultaneously, they recognized two subgroups in this family according to the differences of the mandibular molar shape: the *Scopelocheirus* group, which has a columnar molar bearing a small triturative surface and the *Paracallisoma* group, in which the molar is a non-setose triangular flap or is absent (Lowry and Stoddart 1997). Kilgallen and Lowry (2015) reviewed the worldwide species of Scopelocheiridae and the two subgroups mentioned by Lowry and Stoddart (1997) were ranked as subfamily level: Scopelocheirinae and Paracallisominae. The Scopelocheirinae is a small group including only eight species of three genera (Kilgallen and Lowry 2015). The genus *Aroui* Chevreux, 1911 included in the Scopelocheirinae is characterized from other Scopelocheiridae genera by bearing unusually long and distally barbed setae on the outer plate of maxilla 2. There are only four valid species worldwide: *Aroui americana* Lowry & Stoddart, 1997 from the Gulf of Mexico and Argentina; *Aroui hamatopodus* Lowry & Stoddart, 1989 from Australia; *Aroui onagawae* (Takekawa & Ishimaru, 2000) from Japan; and *Aroui setosus* Chevreux, 1911 from the Mediterranean Sea (Chevreux 1911, Lowry and Stoddart 1997, 1989, Takekawa and Ishimaru 2000, Kilgallen and Lowry 2015). However, hitherto the Scopelocheiridae has not been recorded from Korean waters. In this study, a new scopelocheirid lysianassoid, *Aroui minusetosus* sp. n., is reported with detailed description and illustrations, and a key to all known species of the genus *Aroui* is provided.

## Materials and methods

The collected specimens of lysianassids were initially fixed in 80% ethyl alcohol in the field and then preserved in 95% ethyl alcohol after sorting in the laboratory. Specimens were stained with lignin pink before dissection. Their appendages were dissected in petri dishes or excavated microscopic slides filled with mixed solution of glycerol-ethanol using dissecting forceps and needles under a stereomicroscope (Leica M205), and mounted onto temporary slides using glycerol. To prepare illustrations, pencil drawings were made under a light microscope (Leica DMLB) with the aid of a drawing tube. These were then scanned, digitally inked, and arranged on digital plates using the methods described by Coleman (2003, 2009). Definition of the term for ‘seta’ and its types follows that of Watling (1989). Examined specimens were deposited in the collection of the National Institute of Biological Resources (**NIBR**) of Korea.

## Systematic account

### Order Amphipoda Latreille, 1816

#### Superfamily Lysianassoidea Dana, 1849

##### Family Scopelocheiridae Lowry & Stoddart, 1997

Korean Name: Teol-son-gin-pal-yeop-sae-u-gwa, new

###### Subfamily Scopelocheirinae Kilgallen & Lowry, 2015

Korean Name: Teol-son-gin-pal-yeop-sae-u-a-gwa, new

####### Genus *Aroui* Chevreux, 1911

Korean Name: Teol-son-gin-pal-yeop-sae-u-sok, new

######## Aroui minusetosus sp. n.

Taxon classificationAnimaliaAmphipodaLysianassoidea

http://zoobank.org/0DE2B629-ED1F-46EB-883C-EFDF8196AC78

**Figure 1.**
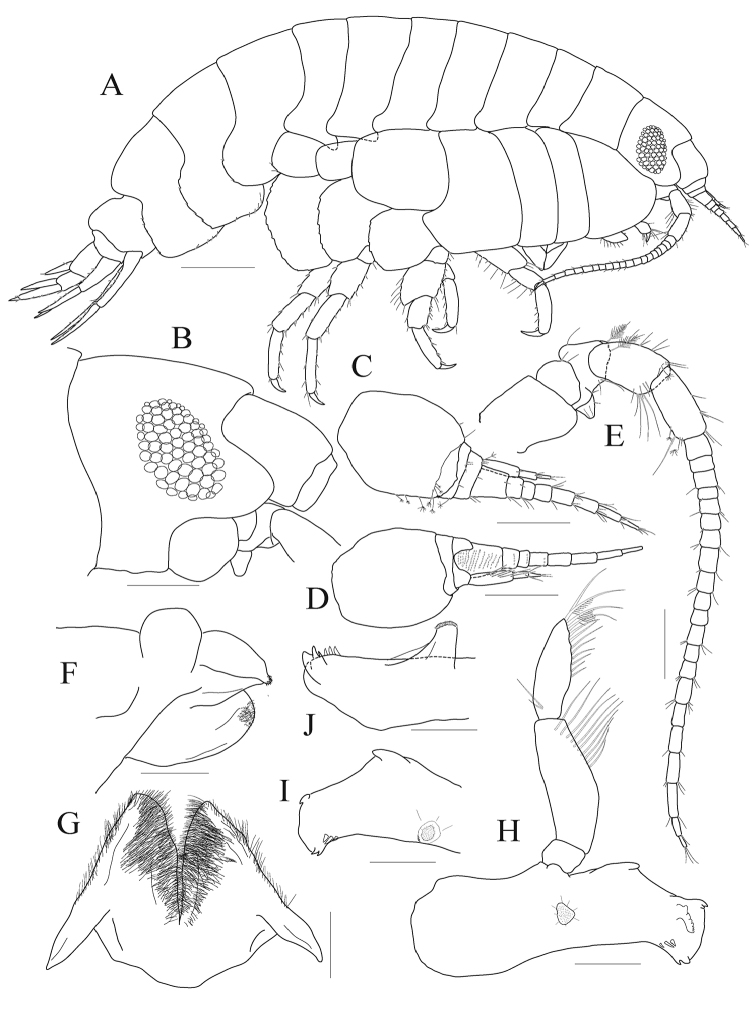
*Aroui minusetosus* sp. n., holotype male, NIBRIV0000806536, 4.3 mm. **A** habitus **B** head **C** antenna 1, lateral **D** antenna 1, medial **E** antenna 2 **F** upper lip, lateral **G** lower lip **H** left mandible **I, J** right mandible. Scale bars: 0.1 mm (**F–J**), 0.2 mm (**B–E**), 0.5 mm (**A**).

**Figure 2.**
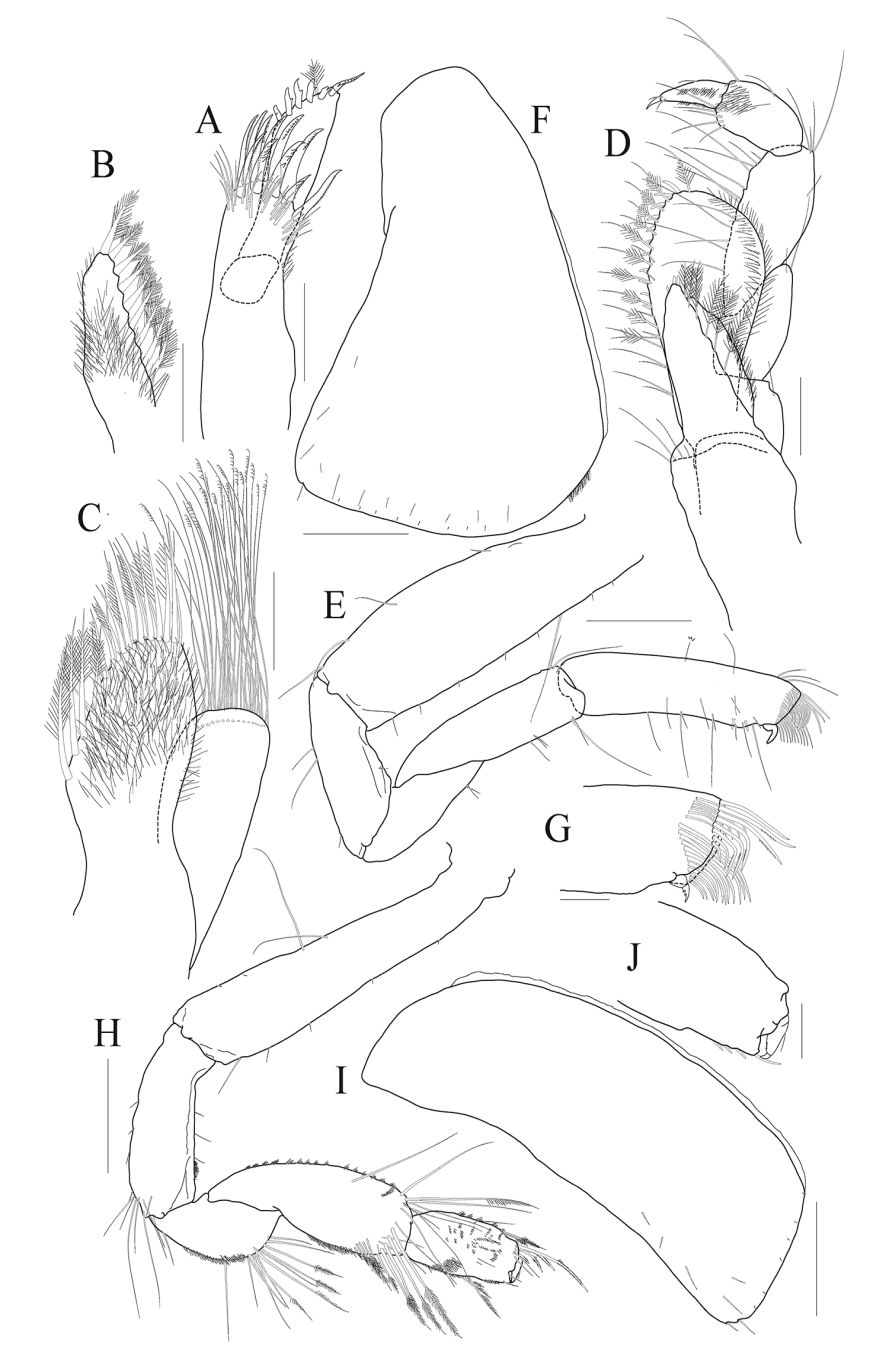
*Aroui minusetosus* sp. n., holotype male, NIBRIV0000806536, 4.3 mm. **A** maxilla 1 **B** inner plate of maxilla 1 **C** maxilla 2 **D** maxilliped **E** gnathopod 1 **F** coxa 1 **G** palm and dactylus of gnathopod 1 **H** gnathopod 2 **I** coxa 2 **J** palm and dactylus of gnathopod 2. Scale bars 0.05 mm (**G, J**), 0.1 mm (**A–D**), 0.2 mm (**E, F, H, I**).

**Figure 3.**
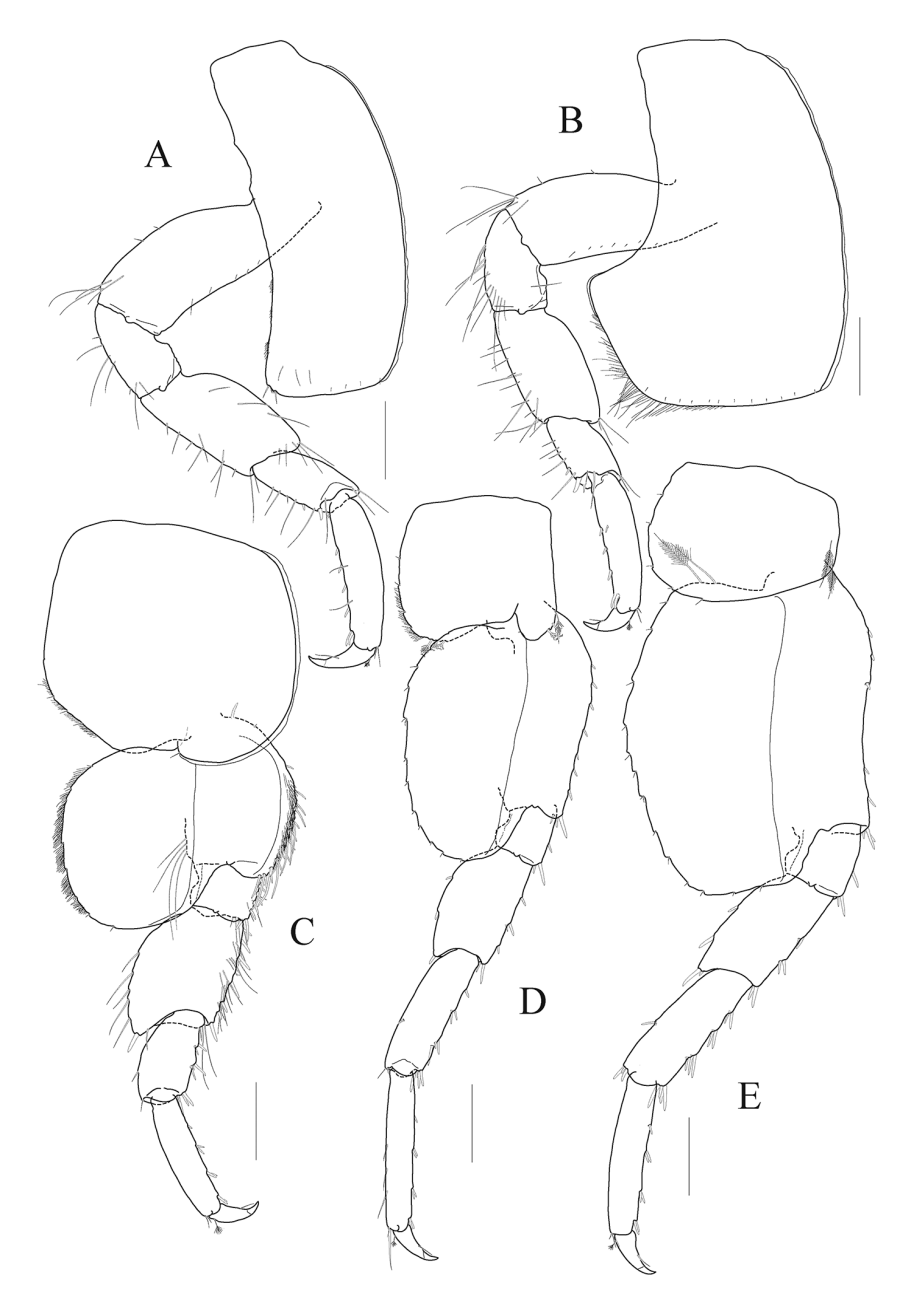
*Aroui minusetosus* sp. n., holotype male, NIBRIV0000806536, 4.3 mm. **A** pereopod 3 **B** pereopod 4 **C** pereopod 5 **D** pereopod 6 **E** pereopod 7. Scale bars 0.2 mm (**A–E**).

**Figure 4.**
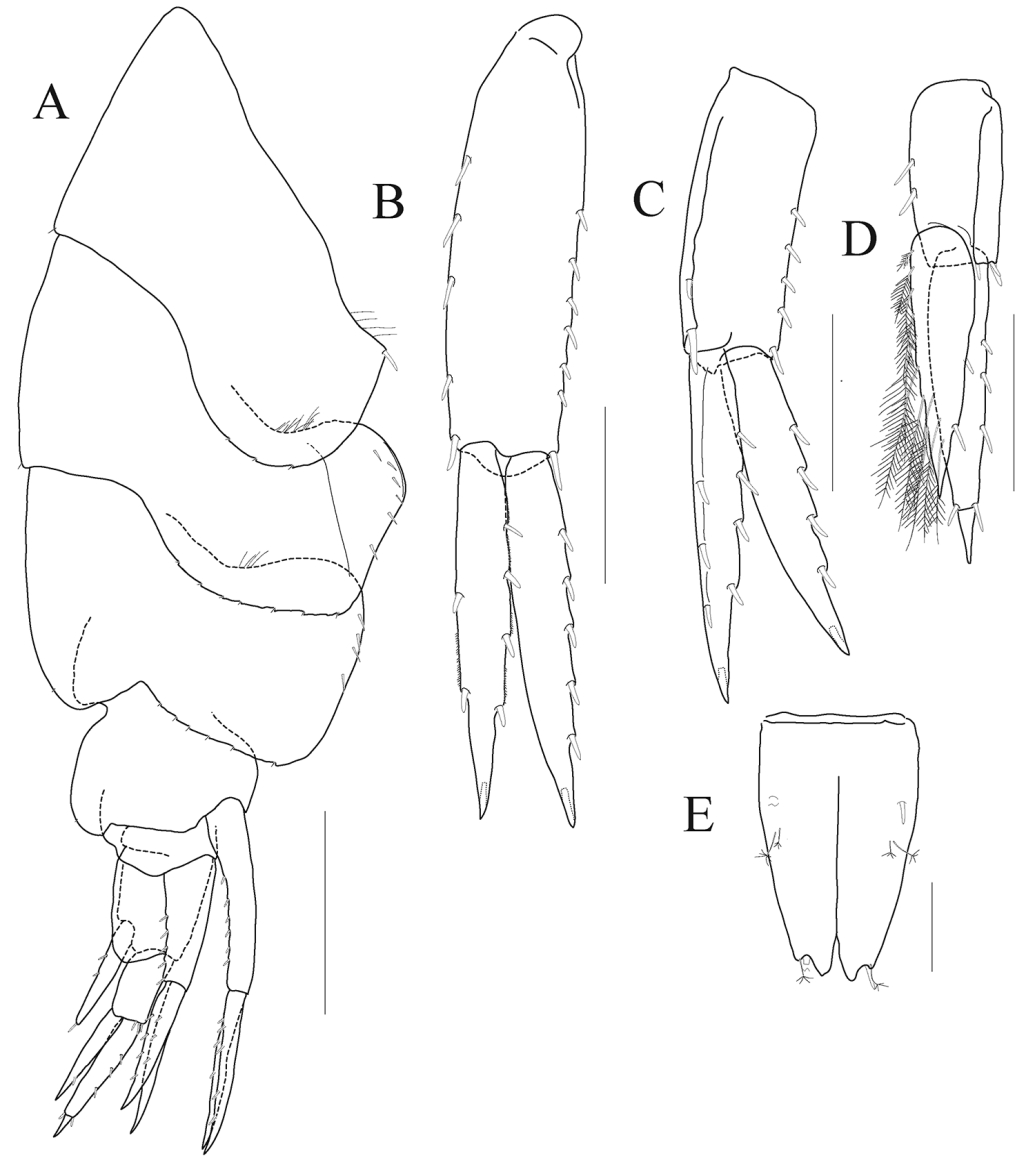
*Aroui minusetosus* sp. n., holotype male, NIBRIV0000806536, 4.3 mm. **A** pleon, lateral **B** uropod 1 **C** uropod 2 **D** uropod 3 **E** telson. Scale bars 0.1 mm (**E**), 0.2 mm (**B–D**), 0.5 mm (**A**).

Figs 1 , 2 , 3 , 4 Korean Name: Teol-son-gin-pal-yeop-sae-u, new

######### Type locality.

Somaemul Island (34°37.656'N, 128°32.467'E, depth 52 m), Gyeongsangnam-do, South Korea.

######### Material examined.

Holotype: adult male, 4.3 mm, NIBRIV0000806536. Paratype: one male, 3.0 mm, NIBRIV0000807161; all dissected appendages and remain bodies of type specimens were preserved in 95% ethanol; collected from the type locality at 12 May 2012, by grab sampling. These specimens were provided by Prof. H.-Y. Soh.

######### Etymology.

The composite epithet of the specific name of *minusetosus* is a combination of the Latin *minus* and *setosus*, referring to having less setose appendages.

######### Diagnosis.

Head eyes ovoid, ommatidia large. Antennae calceoli absent. Mandible with columnar molar process, elevated, triturative surface weakly developed. Maxilla 1 inner plate with plumose setae along medial margin and apex; outer plate with toothed setae apically in 7/4 arrangement; palp article 3 swollen distally, with dentate setae apically. Maxilla 2 inner plate longer than outer plate; outer plate with marginal and submarginal rows composed of barbed and simple setae apically (all setae extremely elongate). Coxae 1–3 not densely setose ventrally; coxa 4 setose posteroventrally. Gnathopod 1 scopelocheirin form; coxa 1 subtriangular; propodus slightly longer than carpus, with rows of long setae forming tuft distally, palm absent; dactylus extremely reduced, anchored at posterodistal corner. Gnathopod 2 propodus subrectangular, with four robust setae posterodistally (distal locking seta extremely elongate), palm nearly transverse, with small protrusion. Pereopods 3–4 moderately developed. Pereopod 5 coxa anterior lobe slightly expanded downward than posterior lobe; basis shorter than coxa, wider than long, anterior margin distal 2/3 length with many elongate robust setae marginally and minute setae submarginally, posterior lobe largely expanded; ischium and merus lined with many simple and robust setae anteriorly; merus posterior margin expanded, with slender setae on distal 2/3 length, posterodistal corner produced (reaching 1/3 length of merus) with robust seta. Pereopod 6 longer and slender than pereopod 5; merus about half as long as basis, expanded posteriorly; carpus rectangular, 1.2 × as long as merus; propodus linear, 1.1 × as long as carpus, with simple long setae on distal half of posterior margin. Pereopod 7 slightly longer and stouter than pereopod 6; basis longer than that of pereopod 6; merus posterior lobe weaker, but setae stouter than those of pereopod 6; carpus and propodus stouter than those of pereopod 6. Epimeron 1 anteroventral corner angulate with one robust seta, posterior margin round and with small notches. Epimera 2–3 expanded and with facial setae anteroventrally, posterior margins lined with small notches. Urosomite 1 with deep dorsal depression and mid-dorsal carina. Uropods 1–2 peduncles longer than rami, with robust setae on lateral and medial margins; outer rami with lateral robust setae only. Uropod 3 shorter than uropod 2; outer ramus bi-articulate; inner ramus not reaching distal end of proximal article of outer ramus in position. Telson cleft about 70%, each lobe with deep apical notch bearing one pair of robust and sensory seta, with one robust seta and one pair of sensory setae dorsolaterally.

######### Description of holotype male.

***Head.*** Lateral cephalic lobes expanded anteriorly, round; eyes ovoid, ommatidia large (Fig. 1B).

*Antenna 1* (Fig. 1C, D) 0.6 × as long as antenna 2, as long as head to pereonite 1 combined; peduncular article 1 swollen anteriorly; accessory flagellum 3-articulate; flagellum article 1 distinctly elongate; calceoli absent.

*Antenna 2* (Fig. 1E) 0.3 × as long as body; peduncular articles moderately developed; flagellum 21-articulate; calceoli absent.

*Upper lip* (Fig. 1F) epistome concave, separated from upper lip; upper lip slightly produced, rounded.

*Lower lip* (Fig. 1G) with developed mandibular processes

*Mandible* (Fig. 1H–J) incisor smooth but bearing blunt denticles on both sides; lacinia mobilis present on left mandible only, stemmed, expanded distally, with irregularly cusped blade; three small raker setae present on both mandibles, patch setules between raker setae and molar processes absent; molar process columnar, elevated, with triturative, rather smooth surface; lateral setigerous crest absent; palp attached midway, 3-articulate; article 2 longest, swollen anteriorly, with an oblique row of ten setae distally; article 3 weakly falcate, 0.7 × as long as article 2, with twelve setae from middle of inner margin to apex.

*Maxilla 1* (Fig. 2A, B) inner plate narrowing distally, not short, setose, with nine plumose setae along medial margin and apex; outer plate with eleven toothed setae apically in 7/4 arrangement and with several setae submarginally; palp bi-articulate, distal article swollen distally, apical margin oblique, with six mono-dentate short setae, one multi-dentate elongate seta, and one plumose seta.

*Maxilla 2* (Fig. 2C) each plate broad; inner plate 1.3 × as long as outer plate, with two rows of simple and plumose setae along distal half of medial and apical margins; outer plate with one marginal and one submarginal rows composed of barbed and simple setae apically (all setae extremely elongate).

*Maxilliped* (Fig. 2D) inner plate with one mediodistal row of plumose setae, apex with three nodular setae; outer plate well developed, subovoid, apex reaching the middle of palp article 3, lined with 13 nodular setae, eleven plumose setae, and two pairs of simple setae along mediodistal margin; palp 4-articulate, article 2 2.0 × as long as article 1, article 4 0.7 × as long as article 3, with one short apical seta.

***Pereon.*** *Gnathopod 1* (Fig. 2E–G) scopelocheirin form; coxa 1 large, subtriangular, expanded distally, round ventrally; basis 0.9 × as long as coxa, anterior margin straight, lined with short setae, posterior margin slightly expanded; ischium 0.4 × as long as basis; carpus elongate, 0.7 × as long as basis; propodus subrectangular, 1.1 × as long as carpus, slightly curved, with one lateral and two medial rows of long setae forming tuft distally, palm absent; dactylus extremely reduced, anchored at posterodistal corner.

*Gnathopod 2* (Fig. 2H–J) slender, subchelate; coxa 2 subrectangular, 2.9 × as long as wide, slightly curved posteroventrally, with two small notches posteroventrally; basis 0.9 × as long as coxa, slightly widened distally; ischium elongate, slightly dilated posterodistally, 0.4 × as long as basis; merus 0.6 × as long as ischium, round posteriorly, with many short setae and one cluster of long setae; carpus 0.6 × as long as basis, anterior margin swollen, with several clusters of short setae, longest seta at anterodistal corner reaching distal end of propodus, with two rows of plumose setae posterodistally; propodus subrectangular, 0.6 × as long as carpus, with four robust setae posterodistally (distal locking seta extremely elongate), palm nearly transverse, with small protrusion posterodistally; dactylus falcate, slightly beyond palm.

*Pereopod 3* (Fig. 3A) stout; coxa subrectangular, 3.0 × as long as wide, slightly curved and with two small notches posteroventrally; basis 0.7 × as long as coxa, subtrapezoidal, somewhat expanded posterodistally; ischium elongate, 0.4 × as long as basis, anterior lobe weak; merus expanded anteriorly, anterodistal corner weakly produced; carpus 0.7 × as long as merus, somewhat expanded distally, with simple and robust setae on posterior margin; propodus 1.5 × as long as carpus, lined with robust setae on posterior margin, with one pair of locking setae posterodistally; dactylus falcate, 0.4 × as long as propodus, unguis developed.

*Pereopod 4* (Fig. 3B) coxa deeper than wide, expanded posteroventrally; other articles nearly similar to those of pereopod 3.

*Pereopod 5* (Fig. 3C) coxa large, subquadrate, slightly wider than long, weakly bilobate and anterior lobe slightly expanded downward than posterior lobe, posteroventral margin oblique, lined with minute setae, with three minute notches each bearing seta; basis subovoid, smaller than coxa, wider than long, anterior margin round, with many elongate robust setae marginally and minute setae submarginally on distal 2/3 length, posterior lobe largely expanded, posterodistal end reaching 1/4 length of merus, margin weakly crenulate and lined with minute setae, with one row of four setae medially; ischium and merus lined with many simple and robust setae anteriorly; merus posterior margin expanded, with five slender setae distally on 2/3 length, posterodistal corner produced (reaching 1/3 length of merus) with one robust seta; carpus dilated posterodistally, 0.8 × as long as merus, anterior margin crenulate and lined with robust setae; propodus 1.3 × as long as carpus, with single and paired robust setae on anterior margin; dactylus falcate, 0.4 × as long as propodus, unguis developed.

*Pereopod 6* (Fig. 3D) longer and more slender than pereopod 5; coxa subrectangular, smaller than that of pereopod 5, bilobate, anterior lobe smaller than posterior lobe, expanded downward, with three plumose setae anteriorly, posterior lobe weakly crenulate, with three plumose setae posteroventrally and minute setae on posterior margin; basis ovoid, 1.7 × as long as wide, anterior margin round proximally and remains nearly straight bearing short robust setae, with one cluster of elongate and short robust setae at anterodistal corner, posterior margin well expanded, smooth, weakly crenulate, posterodistal end reaching 1/4 length of merus; merus half as long as basis, expanded posteriorly; carpus rectangular, 1.2 × as long as merus; propodus linear, 1.1 × as long as carpus, with single and paired robust setae on anterior margin and simple long setae on distal half of posterior margin; dactylus falcate, 0.4 × as long as propodus, unguis developed.

*Pereopod 7* (Fig. 3E) 1.1 × as long as and stouter than pereopod 6; coxa unilobate, with two plumose setae anteriorly, dilated and weakly crenulate posteroventrally; basis larger than that of pereopod 6, anterior margin slightly concave distally at 2/3 length, posterior lobe well developed, margin weakly crenulate, with two plumose setae proximally, somewhat flattened and angulate distally; merus posterior lobe weaker, but setae stouter than those of pereopod 6; carpus and propodus stouter than those of pereopod 6; dactylus falcate, 0.4 × as long as propodus, unguis developed.

***Pleon.*** *Epimeron 1* not produced but angulate bearing one robust seta anteroventrally, posterior margin round and with three small notches. *Epimera 2–3* expanded, with facial setae anteroventrally, posterior margins lined with small notches. *Urosomite 1* with deep dorsal depression and mid-dorsal carina (Fig. 4A).

*Uropod 1* (Fig. 4B) longest; peduncle 1.4 × as long as outer ramus, with seven robust setae on dorsolateral margin and five elongate robust setae on dorsomedial margin; outer ramus with five lateral robust setae only; inner ramus 0.9 × as long as outer ramus, with two medial and four lateral robust setae.

*Uropod 2* (Fig. 4C) 0.7 × as long as uropod 1; peduncle with two robust setae medially (distal seta stoutest) and five robust setae laterally on each dorsal margin; outer ramus 1.3 × as long as peduncle, with five lateral robust setae only; inner ramus 1.1 × as long as outer ramus, with four lateral and three medial robust setae.

*Uropod 3* (Fig. 4D) 0.8 × as long as uropod 2; peduncle 0.7 × as long as outer ramus; each ramus with plumose setae on medial margin; outer ramus bi-articulate, distal article 0.2 × as long as proximal article; inner ramus 0.8 × as long as outer ramus, not reaching distal end of proximal article of outer ramus in position.

*Telson* (Fig. 4E) longer than broad, cleft about 70%, each lobe with deep apical notch bearing one robust and one sensory seta apically, with one robust seta and one pair of sensory setae dorsolaterally.

######### Remarks.

The subfamily Scopelocheirinae is a small group of Scopelocheiridae, composed of only eight species in three genera, united by the synapomorphy of the narrow columnar mandibular molar different from other lysianassoids (Kilgallen and Lowry 2015). In their review, the diagnosis of *Aroui* was restricted concerning the sharing of the unusual long, distally barbed setae on the outer plate of maxilla 2 (Kilgallen and Lowry 2015). The Korean scopelocheirid specimens in this study also show these synapomorphic characters and they are easily assigned to the genus *Aroui*. This genus has only four valid species: *A. americana* Lowry & Stoddart, 1997; *A. hamatopodus* Lowry & Stoddart, 1997; *A onagawae* (Takekawa & Ishimaru, 2000); and *A. setosus* Chevreux, 1911. *Aroui minusetosus* sp. n. is readily distinguished from *A. americana*, *A. hamatopodus* and *A. setosus* in that only coxa 4 has setose margin posteroventrally (vs. all coxae 1–4 of *A. americana*, *A. hamatopodus* and *A. setosus* are densely setose ventrally). The new species is quite similar to *A. onagawae* from Japan having non-setose coxae 1–3, but show a difference in coxa 4: in *A. minusetosus*, it is densely setose with long setae posteroventrally, but only with a moderate setation posteriorly in *A. onagawae*. On the other hand, this new species has a less dense setation on the whole body compared to the original description of *A. onagawae* (Takekawa and Ishimaru 2000). At first, we considered the much weaker setation related to the developmental stage, but the Korean specimens show an elongated antenna 2 that indicates maturity in male, and the body length of the new species is longer than the type specimen of *A. onagawae* (Takekawa and Ishimaru 2000). Additionally, the new species shows some differences from *A. onagawae* as follows: 1) the eyes are ovoid and their ommatidia are larger in *A. minusetosus* sp. n. (vs. pyriform and smaller ommatidia in *A. onagawae*); 2) antenna 1 flagellum is composed of eight articles in *A. minusetosus* sp. n. (vs. 13 articles in *A. onagawae*); 3) gnathopod 2 is subchelate with a small protrusion on the palm in *A. minusetosus* sp. n. (vs. minutely chelate and without protrusion in *A. onagawae*); 4) gnathopod 2 propodus has a row of four robust setae posterodistally (distal locking seta is elongate and not paired) in *A. minusetosus* sp. n. (vs. having a pair of locking setae in *A. onagawae*); and 5) both telson each lobes have a robust seta dorsally in *A. minusetosus* sp. n. (vs. three dorsal robust setae in *A. onagawae*) (see the Table 1, Takekawa and Ishimaru 2000). This is the first record of a scopelocheirid lysianassoid from Korean waters.

**Table 1.** Morphological differences between worldwide *Aroui* species.

Characters	*A. americana*	*A. hamatopodus*	*A. onagawae*	*A. setosus*	*A. minusetosus*
**Eyes**	poorly developed	well developed	well developed	well developed	well developed
shape	ovoid	ovoid	pyriform	ovoid	ovoid
size of each ommatid	large	moderate	moderate	moderate	large
**Antenna 1**					
flagellum	9-articlulate	9-articlulate	13-articlulate	10-articlulate	8-articlulate
**Gnathopod 1**					
length ratio of carpus and propodus	1.0 : 1.2	1.0 :1.1	1.0 :1.1	1.0 :1.0	1.0 :1.1
dactylus	covered with sensory setae	covered with sensory setae	more reduced, without sensory setae	covered with sensory setae	more reduced, without sensory setae
**Gnathopod 2**					
palm	minutely chelate	minutely chelate	minutely chelate	minutely chelate	subchelate
defining setae	paired	paired	paired	absent	single
**Coxae 1–4**					
coxa 1 anterior margin	convex	convex	straight	concave	convex
ventral margin	densely setose	densely setose	not setose in coxae 1–3 (coxa 4 setose posteriorly)	densely setose	not setose in coxae 1–3 (coxa 4 setose posteroventrally)
**Pereopods 3–7**					
setation	moderate	moderate	densely setose	moderate	moderate
pereopod 5 basis medial row of plumose setae	present	absent	absent	absent	absent
pereopod 6 propodus hooked setae on posterior margin	present	present	present	absent	absent
**Uropod 1** *number of setae (medial + lateral)*					
peduncle	5 + 6	7 + 6	14 + 22	many setae (?)	5 + 7
inner ramus	0 + 3	3 + 4	9 + 11	?	2 + 4
outer ramus	1 + 1	0 + 5	0 + 10	?	0 + 5
**Uropod 2** *number of setae (medial + lateral)*					
peduncle	5 + 3	3 + 8	10 + 15	many setae (?)	2 + 5
inner ramus	2 + 3	5 + 5	12 + 12	3 + 2	3 + 4
outer ramus	0 + 3	0 + 4	0 + 9	0 + 2	0 + 4
**Telson**					
dorsal robust setae	one pair	absent	three pairs	absent	one pair
**Reference**	Lowry and Stoddart (1997)	Lowry and Stoddart (1989)	Takekawa and Ishimaru (2000)	Lowry and Stoddart (1989)	In this study

####### Key to worldwide species of the genus *Aroui* Chevreux, 1911

**Table:** 

1	Coxae 1–4 ventrally setose	**2**
–	Coxae 1–3 ventrally smooth, coxa 4 weakly setose posteriorly or posteroventrally	**4**
2	Pereopod 5 basis with an well-developed row of many plumose setae on medial surface	***A. americana* Lowery & Stoddart, 1989**
–	Pereopod 5 basis with one cluster of several simple setae medially	**3**
3	Coxa 1 anterior margin convex	***A. hamatopodus* Lowry & Stoddart, 1997**
–	Coxa 1 anterior margin concave	***A. setosus* Chevreux, 1911**
4	All appendages densely setose; gnathopod 2 minutely chelate	***A. onagawae* (Takekawa & Ishimaru, 2000)**
–	All appendages less setose; gnathopod 2 subchelate	***A. minusetosus* sp. n.**

## Supplementary Material

XML Treatment for Aroui minusetosus

## References

[B1] Chevreux E (1911) Campagnes de la *Melita*. Les amphipodes d’Algérie et de Tunisie. Mémoires de la Société Zoologique de France 23: 145–285.

[B2] Coleman CO (2003) “Digital inking”: How to make perfect line drawings on computers. Organism, Diversity and Evolution, Electronic Supplement 14: 1–14.

[B3] Coleman CO (2009) Drawing setae the digital way. Zoosystematics and Evolution 85: 305–310. https://doi.org/10.1002/zoos.200900008

[B4] Dana JD (1849) Synopsis of the genera of Gammaracea. American Journal of Science and Arts, Series 2, 8: 135–140.

[B5] Kilgallen NM Lowry JK (2015) A review of the scopelocheirid amphipods (Crustacea, Amphipoda, Lysianassoidea), with the description of new taxa from Australian waters. Zoosystematics and Evolution 91: 1–43. https://doi.org/10.3897/zse.91.8440

[B6] Latreille PA (1816) Amphipoda In: Nouveau Dictionaire d’histoire naturelle, appliquée aux Arts, à l’Agriculture, à l’Économie rurale et domestique, à la Médecine, etc. Par une société de Naturalistes et d’Agriculteurs (2^nd^ edn). Volume 1. Deterville, Paris, 467–469.

[B7] Lowry JK Stoddart HE (1989) The scopelocheirid genus *Aroui* (Crustacea: Amphipoda: Lysianassoidea) with notes on the association between scopelocheirid amphipods, cassid gastropods and spatangoid echinoids. Records of the Australian Museum 41: 111–120. https://doi.org/10.3853/j.0067-1975.41.1989.139

[B8] Lowry JK Stoddart HE (1997) Amphipoda Crustacea IV. Families Aristiidae, Cyphocarididae, Endevouridae, Lysianassidae, Scopelocheiridae, Uristidae. Memoirs of the Hourglass Cruises 10: 1–148.

[B9] Takekawa AT Ishimaru S (2000) A new species of the genus *Scopelocheirus* (Crustacea: Amphipoda: Gammaridea) from Onagawa Bay, northeastern Japan. Zoological Science 17: 681–687. https://doi.org/10.2108/zsj.17.681 18517305 10.2108/zsj.17.681

[B10] Watling L (1989) A classification system for crustacean setae based on the homology concept. In: Felgenhauer B Watling L Thistle AB (Eds) Functional morphology of feeding and grooming in Crustacea. CRC Press, Rotterdam, 15–26.

